# A New Methodology for Predicting Brittle Fracture of Plastically Deformable Materials: Application to a Cold Shell Nosing Process

**DOI:** 10.3390/ma14071593

**Published:** 2021-03-24

**Authors:** Jae Gun Eom, Sang Woon Byun, Seung Won Jeong, Wan Jin Chung, Man Soo Joun

**Affiliations:** 1 MFRC, Research and Development Center, Jinju, Gyeongsangnam-do 52818, Korea; jgeom@afdex.com (J.G.E.); swjeong@afdex.com (S.W.J.); 2 Hanil Forging, Jincheon, Chungcheongbuk-do 27856, Korea; sww@hifg.co.kr; 3 Department of Mechanical System Design Engineering, Seoul National University of Science and Technology, Seoul 01811, Korea; wjchung@snut.ac.kr; 4 Engineering Research Institute, School of Mechanical Engineering, Gyeongsang National University, Jinju, Gyeongsangnam-do 52828, Korea

**Keywords:** brittle fracture, ductile materials, plastic deformation-induced embrittlement, embrittlement-effective strain, cold shell nosing

## Abstract

The traditional theory of ductile fracture has limitations for predicting crack generation during a cold shell nosing process. Various damage criteria are employed to explain fracture and failure in the nose part of a cold shell. In this study, differences in microstructure among fractured materials and analysis of their surfaces indicated the occurrence of brittle fractures. The degree of “plastic deformation-induced embrittlement” (PDIE) of plastically deformable materials affects the likelihood of brittle fractures; PDIE can also decrease the strength in tension due to the Bauschinger effect. Two indicators of brittle fracture are presented, i.e., the critical value of PDIE and the allowable tensile strength (which in turn depends on the degree of PDIE or embrittlement-effective strain). When the maximum principal stress is greater than the latter and the PDIE is greater than the former, our method determines the likelihood of brittle fracture. This approach was applied to an actual cold shell nosing process, and the predictions were in good quantitative agreement with the experimental results.

## 1. Introduction

Crack initiation and growth have been important issues in cold metal forming for many years [1,2]. Traditionally, researchers have focused on ductile fracture, which can be understood based on damage criteria. A number of damage models have been proposed [3,4,5,6,7,8,9,10,11,12,13,14] to understand crack generation and strain softening in metal forming [15,16,17] and structural engineering [18,19]. These models play major roles in predicting fracture phenomena occurring during metal forming and materials testing, and in mechanical structures.

Damage models have been successfully applied to predict tensile strength even after the fracture point [15,17], as well as chevron cracking [15,16,20,21,22]. However, the predicted rate of decrease in tensile load was lower than in experimental tensile tests. Other studies reported unrealistic rates of decrease in tensile load [15,20], implying that purely damage-based approaches are inappropriate. The concordance between critical damage predictions and experimental results are path- and test dependent [1].

Despite the use of various methods, chevron cracking prediction remains a problem. Most studies failed to predict V-shaped cracks, and all studies to date failed to predict the decreasing slope with the increase in radius, especially near the surface of the extrusion. In addition, the predicted radii of chevron cracks were much smaller than experimentally determined values [22]. These observations suggest that embrittlement due to compressive plastic deformation around the extrusion surface must exert an effect. One major complicating factor is the Bauschinger effect, which may alter plastic deformation behavior to some extent. For example, it can cause almost perfectly plastic materials to behave like strain-hardening materials and vice versa [23].

It is often difficult to determine the reasons for fracture occurrence during cold forging, because the inherent behaviors of materials change with plastic deformation; only the mass remains unchanged. Brittle fracture of ductile materials during cold metal forming is particularly problematic. The degree of “plastic deformation-induced embrittlement” (PDIE) can be increased by embedded inclusions, such as nitrogen and hydrogen as well as detrimental metallic or non-metallic inclusions, in plastically deformable bodies. Kim et al. [24] investigated the effects of nitrogen on the likelihood of fracture in cold forging and reported that it was necessary to minimize nitrogen to prevent the material from cracking. Thomson [25] proposed a physical model of fracture featuring a brittle crack embedded in a plastically deformed medium and extended it to the case of hydrogen embrittlement in steel. Singh et al. [26] studied the effects of non-metallic inclusions on crack formation in forged steel components using various metallurgical techniques and showed that such inclusions can be a major source of brittle fracture in cold forging.

In cold metal forming, brittle fracture can occur during plastic deformation. Brittle fracture of initially ductile materials with moderate forgeability occurs frequently, e.g., in double cup, forward and backward extrusion. Sljapic et al. [27] studied ductile and brittle fractures occurring during cold forming of brass, and reported ductile fracture of an axisymmetric collar, while a brittle fracture was observed in hexagonal-shaped bars in response to large plastic strain. It was concluded that a single fracture criterion cannot explain these fracture cases.

The brittle facture of various materials has been studied theoretically and metallurgically. Jokl et al. [28] studied brittle fracture of a crystalline solid capable of being plastically deformed, taking into consideration the energy consumed by bond stretching and breaking, and by dislocation emission from the crack tip. There have also been studies [29,30] on the phase field theory of brittle fracture, and this remains a topic of significant research interest. The above studies focused on microscopic examination of crack propagation, and did not address increases in the PDIE of ductile materials or the sudden occurrence of brittle fracture. Watanabe et al. [31] proposed a modified Freudenthal damage model, i.e., an energy model, to understand brittle fracture occurring during cold forging of hollow shafts of the cold forgeable material, S48C, when the material had not been subjected to proper heat treatment.

Here, a practical, macroscopic approach for predicting brittle fracture during cold forging of ductile materials is presented. We assumed that the degree of PDIE is isotropic, and also that fracture occurs in a plane normal to the direction of the maximum principal stress when its value exceeds the weighted tensile strength of the material embrittled by compressive plastic deformation.

## 2. Problem Description

**Figure 1:**
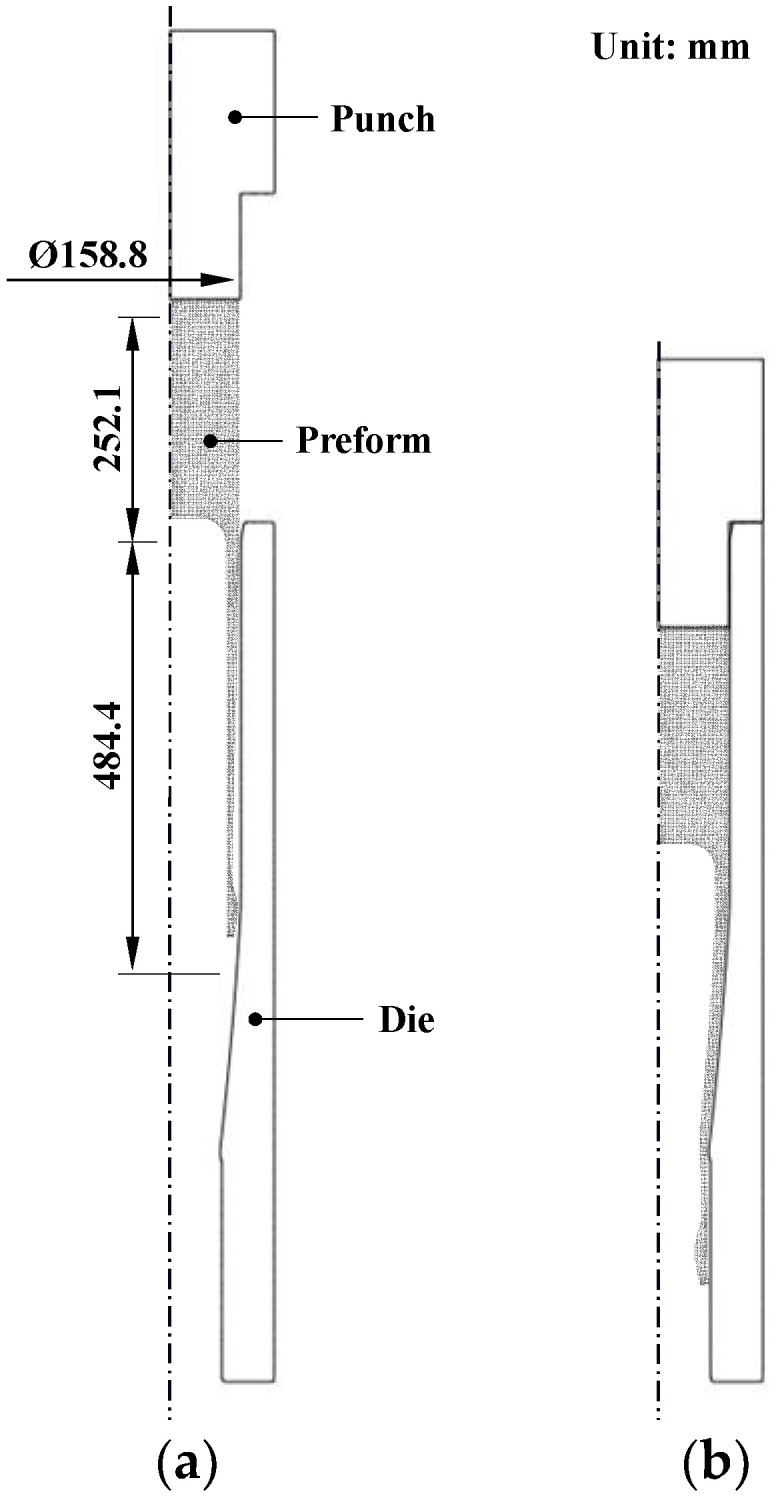
The cold shell nosing process: (**a**) initial configuration and (**b**) final configuration.

Figure 1 shows the cold shell nosing process, which is a special type of cold forging process. The material is AISI 9260 (C: 0.60 wt.%, Si: 1.86 wt.%, Mn: 0.81 wt.%, P: 0.013 wt.%, S: 0.014 wt.%, Al: 0.011 wt.%, Cr: 0.12 wt.%, Mo: 0.03 wt.%, Ni: 0.08 wt.%, Fe: Bal.). The preform shown in Figure 1a is hot forged, spheroidized, machined, and lubricated for the single-stage cold shell nosing process shown in Figure 1b. A hydraulic press was employed for pilot manufacturing.

**Figure 2:**
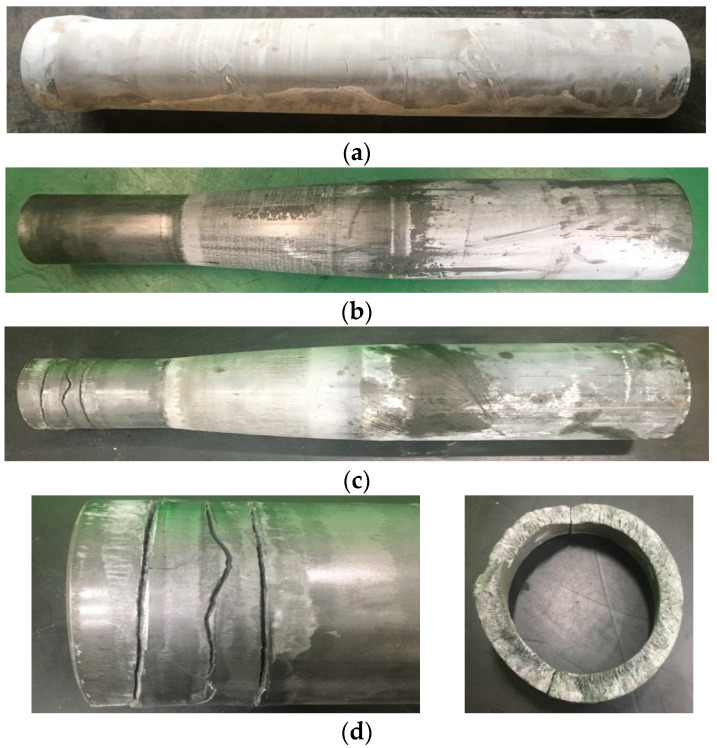
Materials in the cold shell nosing process experiments: (**a**) preform, (**b**) good-quality case, (**c**) fracture case, and (**d**) Expanded view of the fracture.

Figure 2 shows the materials in the cold shell nosing process experiments. Figure 2a shows the lubricated preform, and Figure 2b,c compares good-quality forging and material fracture cases. The fracture rate among all test products was ~3%. The fracture patterns were almost the same as those shown in Figure 2c.

**Figure 3:**
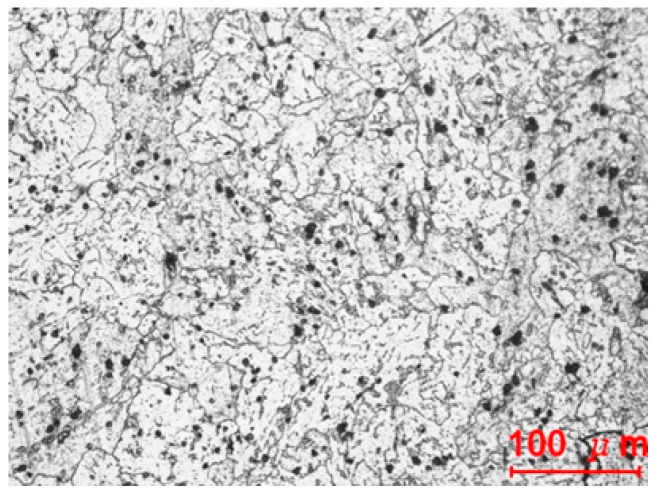
Microstructure of the fractured material.

The microstructure of the fractured material was characterized by a typical spheroidized pearlite, as shown in Figure 3. Visual investigation of the fracture in Figure 2d indicates no characteristics of ductile fracture, i.e., history of damage evolution.

**Figure 4:**
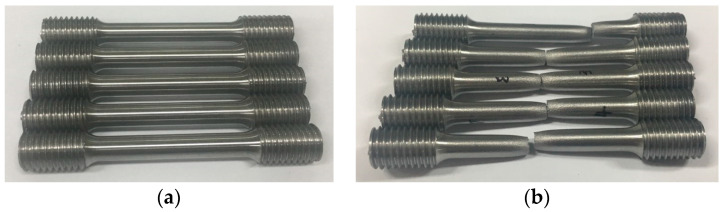
Tensile specimens: (**a**) before tensile test and (**b**) after tensile test.

To determine the causes of fracture, the materials were examined macroscopically and microscopically. The five tensile specimens shown in Figure 4 were fabricated from the preforms, ready to be shell-nosed and pulled by a universal testing machine. The diameter and gauge length were 6 and 30 mm, respectively.

**Figure 5:**
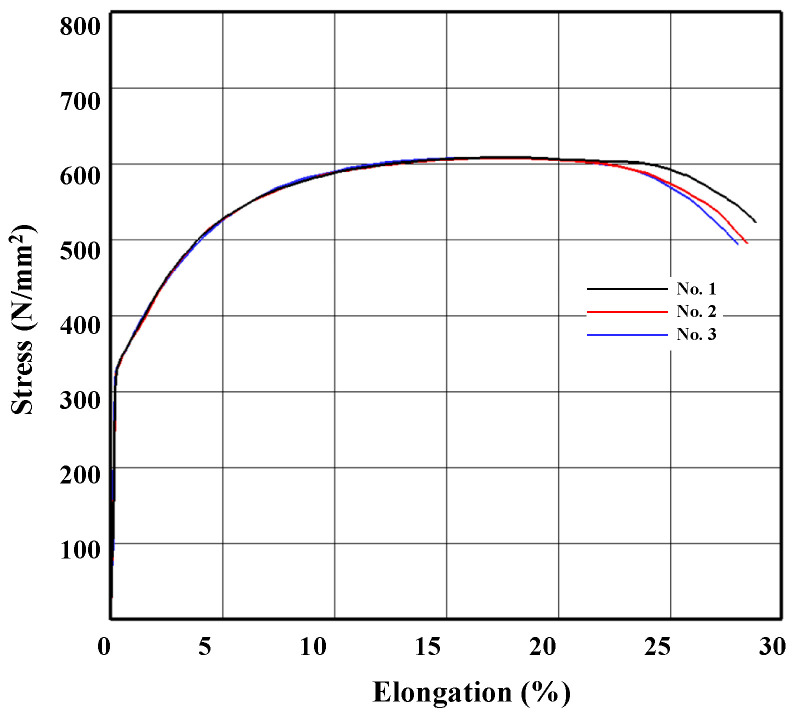
Tensile test.

**Figure 6:**
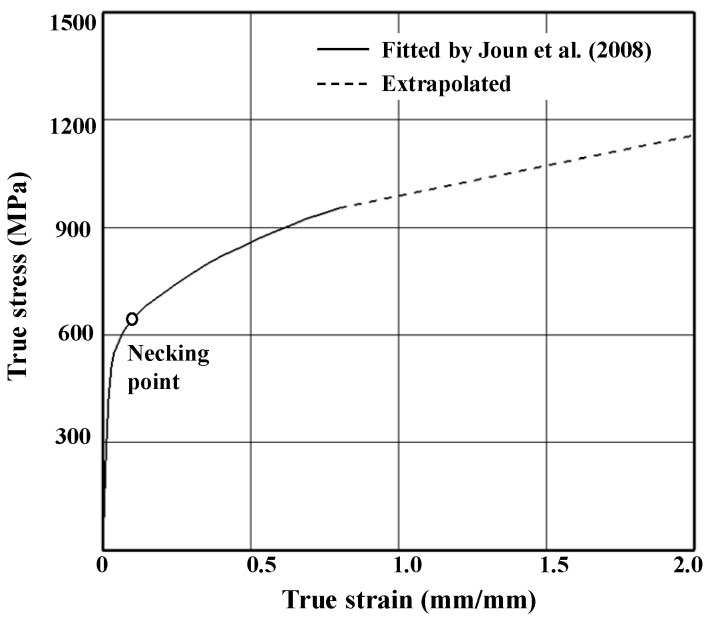
Flow stress determined from the tensile test.

**Figure 7:**
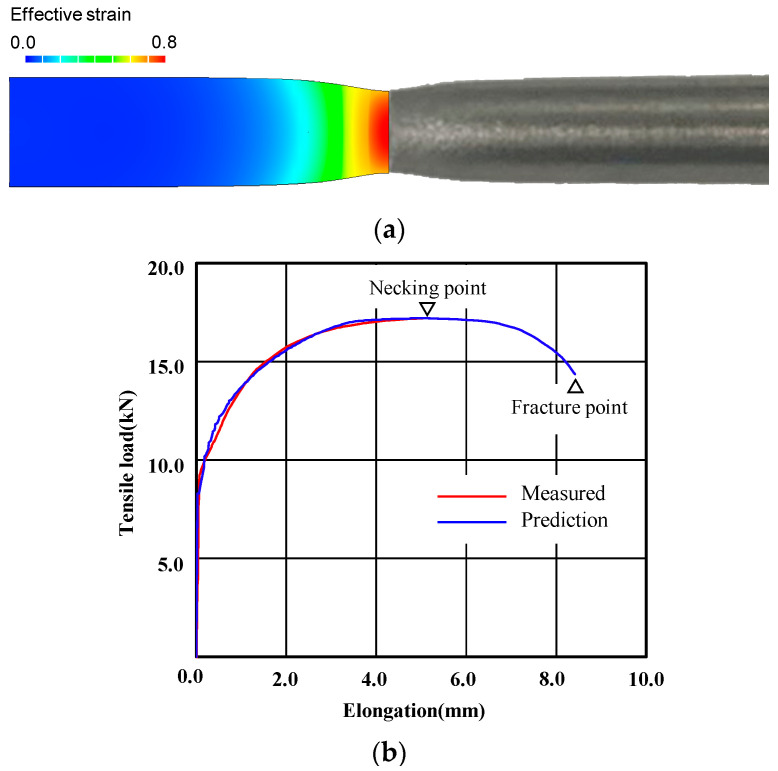
Comparison of predicted and experimental tensile test results: (**a**) shape after the fracture and (**b**) tensile load-elongation curve.

We analyzed the representative elongation-tensile load curve denoted by No. 2 in Figure 5 to determine flow stress using a material identification technique [32]. Flow stress for strain values up to 0.8 was acquired and extrapolated to larger strains, as can be seen in Figure 6. The strain up to which the flow stress was theoretically obtained was quite large. The technique can predict the flow stress at the strain of around 1.5, depending on the materials [33,34,35]. Note that the point marked “necking point” in the true stress-strain curve in Figure 6 corresponds to the actual necking point in Figure 7.

Figure 7 compares necked-shaped tensile specimens (Figure 7a) and the experimental tensile load-elongation curve (Figure 7b) with the tensile test predictions made based on the flow stress shown in Figure 6, with an emphasis on post-necking strain hardening. The comparison implies that the flow stress in Figure 6 is acceptable, especially the flow stress at large strain after the necking point; the experimental and predicted shapes of the specimen after fracture are in good agreement with each other. Notably, the true strain at the necking point, i.e., 0.13, is quite small compared with the maximum strain of 0.8 in the tensile test in the present study. Before the necking point, some discrepancies between the experimental and predicted tensile load-elongation curves can be seen in Figure 7b. However, they are very close to each other after the necking point, which can be accurately predicted regardless of the error [36]; this suggests that the almost-uniform cross-section of the specimen was maintained up to the necking point and that the effect of the error could therefore be neglected when predicting the critical damage arising from the tensile test because damage accumulation is markedly affected by the strain after the necking point.

We simulated the same tensile test to determine the critical damage values of various damage models, based on the maximum damage calculated at the fracture point, including those of Freudenthal [3], McClintock [4], Cockcroft-Latham (original and normalized; CL and NCL, respectively) [5], Rice and Tracey (RT) [6], Brozzo et al. (BDR) [7], Norris et al. (NRMQ) [8], Oyane et al. (OSOS) [9], Chaoudadi et al. (CMV) [10], Rice et al. (Rice-Tracey-Cockroft-Latham; RTCL) [11], Ko et al. (KH) [12], Bai and Wierzbicki (BW) [13] for both unnormalized lode angles (BWUL) and normalized lode angles (BWNL), and Lou and Huh (LH) [14]. The material constants for all models are given in Table 1, along with their associated model equations and references. $\sigma_{m}$, $\bar{\varepsilon }$ and $\sigma_{i}$($\sigma_{1}\geq \sigma_{2}\geq \sigma_{3}$) mean mean stress, effective strain and principal stress, respectively. Note that some of the material constants in Table 1 are not specifically for AISI 9260, because their acquisition is costly and time consuming and the goal of this study was not direct comparison of the damage models, but rather to determine whether the damage models can be used to predict fracture occurring during the cold shell nosing process, as shown in Figure 2d.

**Figure 8:**
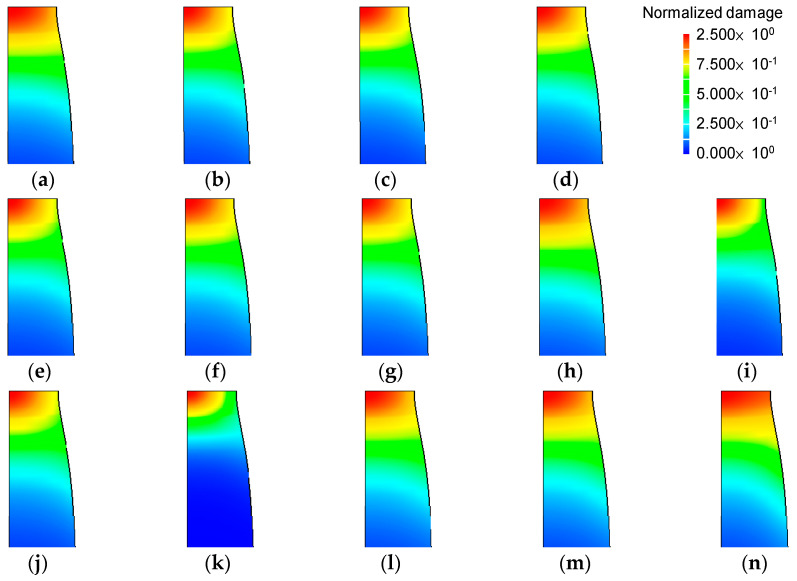
Prediction of normalized damage around the necking point at the fracture instant: (**a**) Freudenthal, (**b**) McClintock, (**c**) original Cockcroft-Latham (CL) (**d**) normalized Cockcroft-Latham(NCL), (**e**) Rice and Tracey (RT), (**f**) Brozzo et al. (BDR), (**g**) Norris et al. (NRMQ), (**h**) Oyane et al. (OSOS), (**i**) Chaoudadi et al. (CMV), (**j**) Rice et al. (Rice-Tracey-Cockcroft-Latham) (RTCL), (**k**) Ko et al. (KH), (**l**) Bai and Wierzbicki both unnormalized lode angles (BWUL), (**m**) Bai and Wierzbicki normalized lode angles (BWNL) and (**n**) Lou and Huh (LH).

The predicted critical damage values, which mean maximum damage values at the necking point at the fracture instant (See Figure 7a), are summarized in Table 1 and the normalized damage values around the necking region at the fracture instant, defined as the calculated damage values divided by their corresponding critical damage values, are shown in Figure 8. Note that the critical damage values were determined by the maximum damage values at the fracture instant and that the normalized damage values at the necking point in Figure 8 are thus all unity. Figure 8 shows that all of the damage models tested predicted the same initial fracture point, i.e., the center of the necking point, even though the distribution of normalized damage can be categorized into three different groups (first group, Freudenthal, McClintock, CL, RT, BDR, NRMQ, and LH; second group, NCL, OSOS, RTCL, and BWUL; and third group, CMV and KH).

## 3. Checking for Ductile Fracture

**Figure 9:**
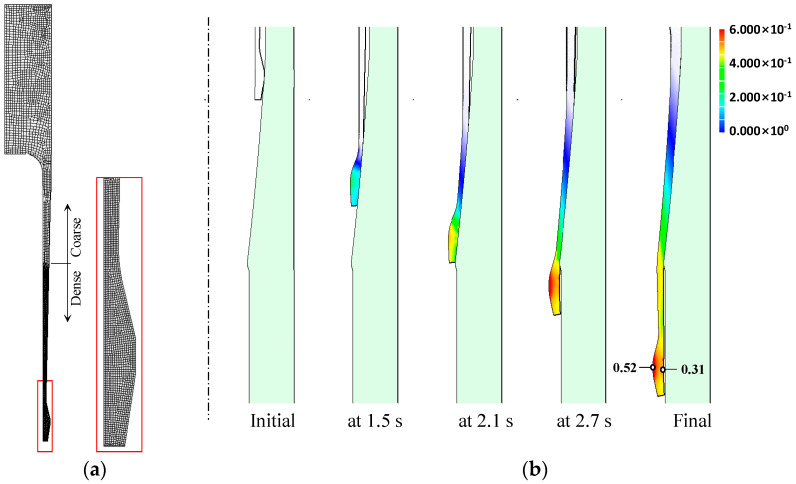
FE (Finite Element) analysis model and history of deformation: (**a**) FE analysis model and (**b**) effective strain distribution.

We simulated the cold shell nosing process using an elastoplastic finite element method [37,38] with all the information outlined in the previous section. We assumed that the material is rate-independent and that the punch velocity was fixed at −1 mm/s. Due to the well-lubricated material surface, the coefficient of friction was assumed to be 0.05. The finite element model was purposely meshed for precise simulation of the nose part, as shown in Figure 9a, because most plastic deformation is concentrated in this region. The deformation over time with effective strain is shown in Figure 9b, indicating that the thick nose part slid down along the die wall with the reduction of its radius under compressive stress; it passed the die orifice between 2.1 and 2.7 s. The maximum effective strain of 0.52 occurred on the internal bulged surface. However, the effective strain was relatively low on the opposite side, i.e., 0.31.

**Figure 10:**
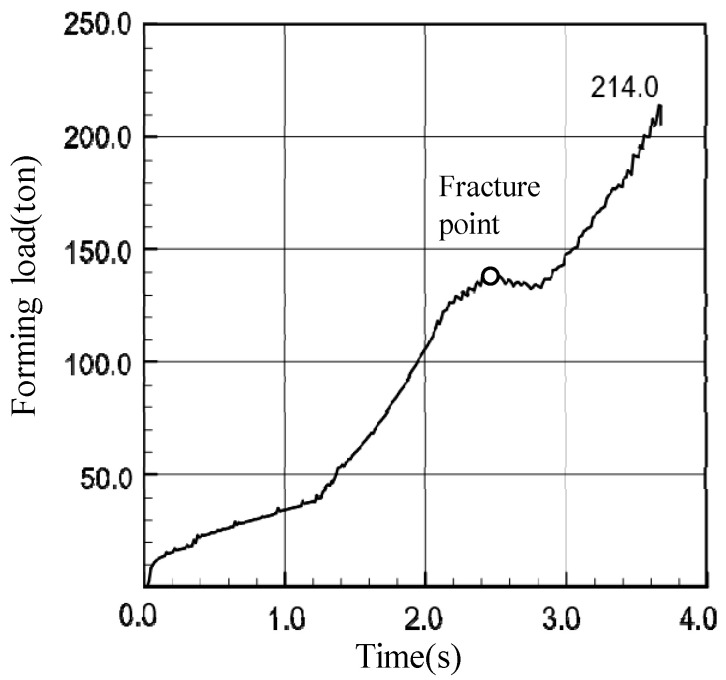
Forming load-time curve.

The predicted forming load-time curve is shown in Figure 10. The forming load increased steadily up to the stroke when the nose part started to separate from the die while oscillating due to intermittent node detachment from the die. While the thick nose part passed the die orifice, the forming load increased to its local maximum, as indicated in Figure 10. From the stroke where the upper side of the material started to show plastic deformation, it increased steadily up to 214 tons. It is interesting to note that the decrease in forming load with stroke increase occurred near the instant of fracture, as shown in Figure 10, which can have direct or indirect effects on the fracture.

**Figure 11:**
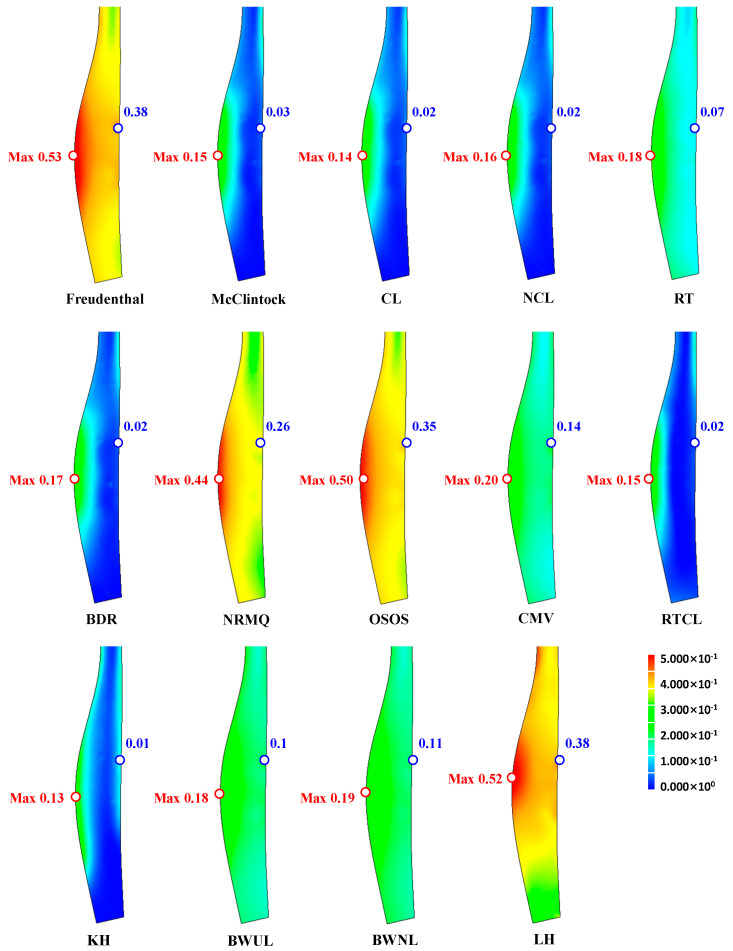
Predictions of normalized damage.

We determined the damage values for all models and then normalized them by dividing by the critical damage. Figure 11 compares the predicted normalized damages in the nose part; the maximum values are listed in Table 2. All of the predicted normalized damage values were much lower than unity. In addition, all of the damage models predicted low damage on the surface of the fractured side, i.e., on the outer diameter. Therefore, we concluded that the actual fracture surface is unrelated to the ductile fracture.

## 4. New Approach to Brittle Fracture

Plastic deformation of material under compression results in the loss of a great deal of tensile strength capacity because of the Bauschinger effect; the material becomes brittle to some extent, depending on the material properties and magnitude of the plastic deformation [39,40]. When a plastically compressed material that has lost a considerable amount of its yield strength is elongated, the material may yield or fracture, even if the tension stress is smaller than the original yield strength or fracture stress. In the case of brittle fracture, the fracture surface is almost normal to the axis of maximum principal stress.

**Figure 12:**
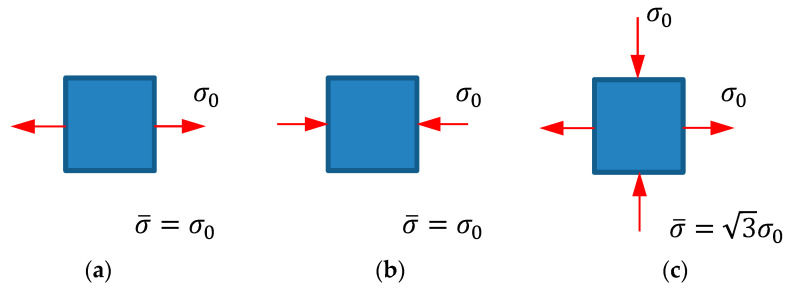
Loading in the plane stress case: (**a**) pure tension, (**b**) pure compression, and (**c**) zero mean stress.

In the case of multiaxial stress and strain, we have to measure the “embrittlement-effective strain” to determine the degree of PDIE of the materials; this is done using a form of scalar function. For the three plane stress cases shown in Figure 12, we present the following compressive strain weighting index (CSWI), ξ, to exclude the ductile fracture-related strain from the multiaxial stress or strain:

(1) $$\xi =\;\;\;\;<1-\xi_{0}<\gamma >>,$$

where <x> is a mathematical operator taking a larger value between zero and x, and γ is defined by

(2) $$\gamma =\;\;\frac{\sigma_{1}}{\bar{\sigma }},$$

where $\sigma_{1}$ and $\bar{\sigma }$ are the maximum principal stress and effective stress, respectively. $\xi_{0}$ in Equation (1) can be dealt with as a material constant. When we assume that $1-\xi_{0}<\gamma >$ is non-negative in the state of non-positive mean stress in the plane stress case, $\xi_{0}\leq \sqrt{3}$ should be satisfied. Based on the zero mean stress case in Figure 12c, we assumed that $\xi_{0}=\sqrt{3}$ for the zero-value condition of CSWI at zero mean stress.

Next, we checked the three special cases, $\gamma =\;\;1,\;\;-1\;\;\mathrm{and}\;1/\sqrt{3}$, with respect to the cases in Figure 12a–c, respectively. Substituting these γ-values into Equation (1), we obtained weighting indices of 0, 1, and 0 for the three special cases, respectively. Note that the effects on fracture of the stresses shown in Figure 12a,c are fully accounted for by the damage model of ductile fracture, implying that the CSWI is able to reflect brittle fracture.

With respect to the time parameter, the strain rate multiplied by the CSWI describes a type of compressive strain, i.e., embrittlement-effective strain, which is calculated as

(3) $$\varepsilon_{B}=\;\;\int_{0}^{t}\xi \;\dot{\bar{\varepsilon }}\;dt,$$

where $\varepsilon_{B}$ is defined as the degree of PDIE, which is used to evaluate the likelihood of brittle fracture. As the ductility continues to govern the material when the degree of PDIE is small, we have to assume the critical value of PDIE $\varepsilon_{B.Cr}$, which is used to determine the conditions necessary for brittle fracture of the plastically compressed material.

In addition, we adopted another weighting function, denoted as $\zeta (\varepsilon_{B})$, called embrittlement function, to reduce the fracture stress based on the degree of PDIE and considering the Bauschinger effect, as follows

(4) $${\bar{\sigma }}_{BF}=\;\;\zeta (\varepsilon_{B}){\bar{\sigma }}_{\max},$$

where ${\bar{\sigma }}_{BF}$ is the maximum allowable principal stress for the plastically compressed material and ${\bar{\sigma }}_{\max}$ is the maximum effective stress experienced. In this study, this weighting function was given by the following linear function:

(5) $$\zeta (\varepsilon_{B})=1-B\varepsilon_{B},$$

where B is a material constant, proposed in this study, to reflect the effect of the Bauschinger effect on reducing the allowable tensile stress of the embrittled material. Notably, the B-value can be obtained by tensile test of compressed material or bending test.

Therefore, we evaluated the occurrence of brittle fracture when the maximum principal stress $\sigma_{1}$ exceeded ${\bar{\sigma }}_{\max}$ multiplied by the embrittlement function. As $\sigma_{1}$ keeps to change with the stroke in the state of failure if a function of crack generation is not adopted, the number of solution steps for such failures should be counted to estimate the potential for brittle fracture.

**Figure 13:**
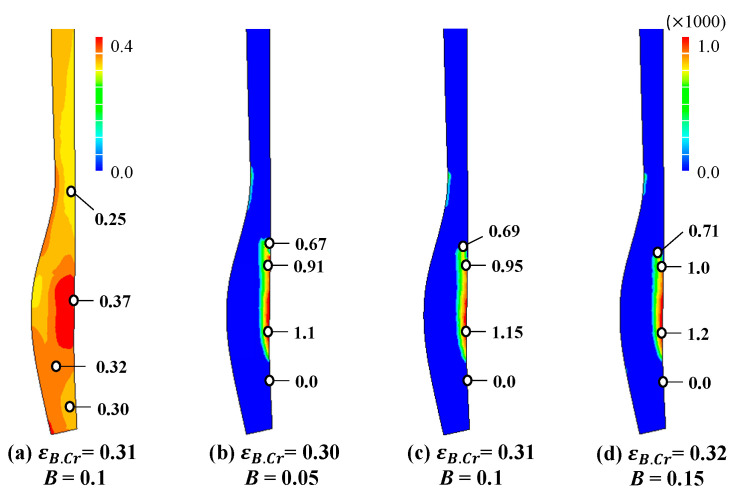
Degree of PDIE (plastic deformation-induced embrittlement) and prediction of brittle fracture. (**a**) Degree of PDIE, (**b**–**d**) brittle fracture maps.

Figure 13a shows the predicted compressive strain, i.e., the degree of PDIE when $\varepsilon_{B.Cr}$ = 0.31 and B = 0.1, while Figure 13b–d shows the brittle fracture maps when $\varepsilon_{B.Cr}$ = 0.30 and B = 0.05, $\varepsilon_{B.Cr}$ = 0.31 and B = 0.1, and $\varepsilon_{B.Cr}$ = 0.32 and B = 0.15, respectively. Comparison of Figure 2 with Figure 13b–d indicates that they are in good agreement with each other in terms of the location of the brittle fracture. Of course, the axis of maximum principal stress was approximately normal to the actual fracture surface.

Note that the critical value of PDIE, denoted by $\varepsilon_{B.Cr}$, calculated qualitatively from the comparison between the experiment in Figure 2c and the predicted degree of PDIE in Figure 13a. At this moment, the quantitative interpretation or determination of the parameters could not be made because we did not couple the crack generation with this approach. Nonetheless, the predicted brittle fracture is greatly meaningful to avoid such a possible fracture in the process design stage, as can be seen from the comparison of Figure 2 and Figure 13.

**Figure 14:**
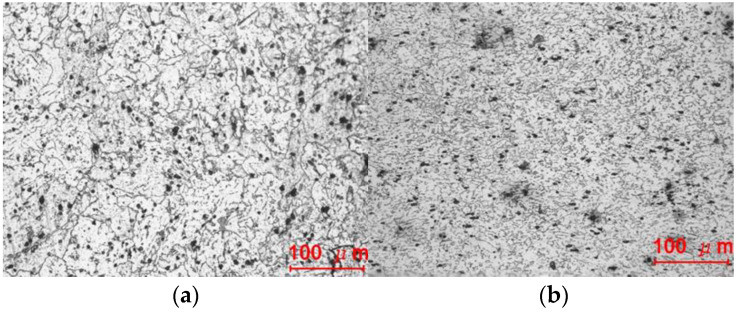
Microstructure of materials: (**a**) fractured and (**b**) non-fractured.

Now, we consider the reason for 3% rate of crack occurrence. Figure 14 compares the microstructures of fractured and non-fractured materials. The fractured material is a typical mixture of pearlite and spheroidite with larger grains, which decreases ductility [41,42,43,44]. This is the same as the case reported by Watanabe et al. [31], indicating that heat treatment of the low-ductility material should be applied carefully, especially when the material is exposed to major changes in stress state after considerable plastic deformation. This type of brittle fracture may occur frequently at corners, where there is no die contact after severe plastic deformation, and under forward and backward extrusion. In all such cases, marked changes are seen in the stress state after severe plastic deformation due to compressive stress. The approach introduced herein is appropriate to predict the possibility of such brittle fractures during cold forging.

## 5. Conclusions

The reasons for fracture in the nose part during the single-stage cold shell nosing process were examined based on the theory of ductile fracture, using various damage models, i.e., the Freudenthal, McClintock, CL and NCL, RT, BDR, NRMQ, OSOS, CMV, RTCL, KH, BWUL and BWNL, and LH models, after tensile testing for detailed investigation of the flow stress and ductile fracture behaviors of the materials. However, the models could not predict fracture around the nose part.

In this study, a practical methodology for predicting brittle fracture was presented based on the degree of PDIE of plastically deformable materials. A new concept, i.e., the CSWI, was proposed to calculate the embrittlement-effective strain, excluding the damage-effective strain from the multiaxial stress. The cumulative strain indexed by CSWI was taken to indicate the degree of PDIE, which affects the likelihood of brittle fracture of plastically deformable material, as well as the fracture stress due to the Bauschinger effect. The critical value of PDIE is a material and process parameter. Our methodology estimates the likelihood of brittle fracture only when the maximum principal stress is greater than the fracture stress, and the PDIE is greater than its critical value.

Assuming that the tensile strength decreases linearly with increasing PDIE, our methodology was applied to determine the reasons for fracture in an actual cold shell nosing process. Comparison of the predicted and experimental fracture shape and position indicated good quantitative agreement. Comparison of the microstructures of the materials between the failure and success cases also supported the conclusions of this study.

## Figures and Tables

**Table 1:** Damage models employed in this study and their material constants.

Damage Model	Model Equations	Material Constants	Critical Damage	Reference
Freudenthal	$D=\int^{\;}\bar{\sigma }d\bar{\varepsilon }$		710.2	[3]
McClintock	$D=\int^{\;}\left(\frac{2}{\sqrt{3}\left(1-n\right)}\right)\sin h\left(\frac{\sqrt{3}\left(1-n\right)}{2}\frac{\sigma_{1}+\sigma_{3}}{\bar{\sigma }}\right)+\frac{\sigma_{1}-\sigma_{3}}{\bar{\sigma }}d\bar{\varepsilon }$	*n* = 0.2	1.08	[4]
CL	$D=\int^{\;}\sigma_{1}d\bar{\varepsilon }$		820.5	[5]
NCL	$D=\int^{\;}\frac{\langle \sigma_{1}\rangle }{\bar{\sigma }}d\bar{\varepsilon }$		0.99	[5]
RT	$D=\int^{\;}A\exp\left(\frac{3}{2}\frac{\sigma_{m}}{\bar{\sigma }}\right)d\bar{\varepsilon }$	*A* = 0.427	0.77	[6]
BDR	$D=\frac{2}{3}\int^{\;}\frac{\sigma_{1}}{\sigma_{1}-\sigma_{m}}d\bar{\varepsilon }$		0.99	[7]
NRMQ	$D=\int^{\;}\frac{1}{1-c\sigma_{m}}d\bar{\varepsilon }$	*c* = 0.7	1.32	[8]
OSOS	$D=\int^{\;}\left(1+\frac{\sigma_{m}}{c\bar{\sigma }}\right)d\bar{\varepsilon }$	*c* = 3.9	0.97	[9]
CMV	$D=\int^{\;}\bar{\sigma }\left(1+\frac{3\alpha \sigma_{m}}{\bar{\sigma }}\times \exp\left(\frac{3}{2}\frac{\sigma_{m}}{\bar{\sigma }}\right)\right)d\bar{\varepsilon }$	α = 0.428	1685.6	[10]
RTCL	$D=\int^{\;}\mathrm{SSd}\bar{\varepsilon }$, $SS=\frac{2\left(1+\eta \sqrt{12-27\eta^{2}}\right)}{3\eta +\sqrt{12-27\eta^{2}}},\;\eta =\;\frac{\sigma_{m}}{\bar{\sigma }}$		1.09	[11]
KH	$D=\int^{\;}\frac{\sigma_{1}}{\bar{\sigma }}\left(1+\frac{3\sigma_{m}}{\bar{\sigma }}\right)d\bar{\varepsilon }$		161.4	[12]
BWUL	$D=\int^{\;}\frac{1}{{\bar{\varepsilon }}_{p}^{\mathrm{crit}}}d\bar{\varepsilon }$, ${\bar{\varepsilon }}_{p}^{\mathrm{crit}}={\hat{\varepsilon }}_{f}\left(\eta ,\;\bar{\theta }\right)$		0.46	[13]
BWNL	$D=\int^{\;}\frac{1}{{\bar{\varepsilon }}_{p}^{\mathrm{crit}}}d\bar{\varepsilon }$, ${\bar{\varepsilon }}_{p}^{\mathrm{crit}}={\hat{\varepsilon }}_{f}\left(\eta ,\;\bar{\theta }\right)$		0.33	[13]
LH	$D=\int^{\;}{\left(\frac{2}{\sqrt{L^{2}+3}}\right)}^{a}{\left(\frac{1}{2}+\frac{3}{2}\frac{\sigma_{m}}{\bar{\sigma }}\right)}^{b}d\bar{\varepsilon }$, $L=\frac{2\sigma_{2}-\sigma_{1}-\sigma_{3}}{\sigma_{1}-\sigma_{3}}$	*a* = 6.0 *b* = 0.3	0.94	[14]

**Table 2:** Comparison of predicted maximum normalized damage values.

Damage Model	Maximum Normalized Damage	Damage Model	Maximum Normalized Damage
Freudenthal	0.53	OSOS	0.50
McClintock	0.15	CMV	0.20
CL	0.14	RTCL	0.15
NCL	0.16	KH	0.13
RT	0.18	BWUL	0.18
BDR	0.17	BWNL	0.19
NRMQ	0.44	LH	0.52

## Data Availability

Data is contained within the article.
